# Correction: Analysis of the effects of acoustic levitation to simulate the microgravity environment on the development of early zebrafish embryos

**DOI:** 10.1039/d1ra90003j

**Published:** 2021-01-19

**Authors:** Li Li, Ning Gu, Huijuan Dong, Bingsheng Li, Kenneth T. V. Grattan

**Affiliations:** School of Life Sciences and Technology, Harbin Institute of Technology Harbin 150080 China; State Key Laboratory of Robotics and Systems, Harbin Institute of Technology Harbin 150080 China dhj@hit.edu.cn; State Key Laboratory of Urban Water Resource and Environment, Harbin Institute of Technology Harbin 150090 China; Key Laboratory of UV Light Emitting Materials and Technology Under Ministry of Education, Northeast Normal University Changchun 130024 China libs@nenu.edu.cn; School of Mathematics, Computer Science and Engineering, City, University of London London EC1V 0HB UK

## Abstract

Correction for ‘Analysis of the effects of acoustic levitation to simulate the microgravity environment on the development of early zebrafish embryos’ by Li Li *et al.*, *RSC Adv.*, 2020, **10**, 44593–44600, DOI: 10.1039/D0RA07344J.

The authors regret that the name of one of the authors (Kenneth T. V. Graham) was shown incorrectly in the original article. The corrected author list is as shown above.

The Royal Society of Chemistry apologises for these errors and any consequent inconvenience to authors and readers.

## Supplementary Material

